# Attitudes toward the pandemic and COVID-19 vaccination intention among German university students and the general population: Results from two cross-sectional surveys

**DOI:** 10.3389/fpubh.2022.1098911

**Published:** 2022-12-15

**Authors:** Sabrina Baldofski, Ezgi Dogan-Sander, Sophia E. Mueller, Freia De Bock, Lena Huebl, Elisabeth Kohls, Christine Rummel-Kluge

**Affiliations:** ^1^Department of Psychiatry and Psychotherapy, Medical Faculty, Leipzig University, Leipzig, Germany; ^2^Department of Psychiatry and Psychotherapy, University Leipzig Medical Center, Leipzig University, Leipzig, Germany; ^3^Unit for Health Services Research, Clinic of General Pediatrics, Neonatology and Pediatric Cardiology, Medical Faculty and University Hospital Duesseldorf, Düsseldorf, Germany; ^4^Department for Tropical Medicine, Bernhard-Nocht-Institute for Tropical Medicine and I. Department of Medicine, University Medical Center Hamburg-Eppendorf, Hamburg, Germany

**Keywords:** COVID-19, vaccination intention, COVID-19 vaccination, university students, COVID-19 attitudes, general population

## Abstract

**Background:**

The COVID-19 pandemic has had an impact on nearly all people. Vaccines provide an effective tool to combat the pandemic, however, vaccination hesitancy remains an issue. This study aims to investigate (a) students' attitudes toward the pandemic, (b) potential differences in attitudes between university students and the general population, and (c) to examine predictors of vaccination intention in both samples.

**Methods:**

In this cross-sectional study data from two research projects were analyzed and compared. First, attitudes toward the COVID-19 pandemic in German university students were assessed within a cross-sectional anonymous online survey (March-April 2021, *N* = 5,639) and analyzed quantitatively and also qualitatively (free text field answers examined positive and negative aspects of the pandemic). Second, data from a cross-sectional survey within the COVID-19 Snapshot Monitoring project (COSMO; 29^th^ wave of data collection, December 2020, *N* = 1,387) in the German general population was analyzed. Both samples, were compared in sharedly used variables, regarding attitudes toward the pandemic and vaccination intention, and factors associated with vaccination (logistic regression analyses).

**Results:**

In comparison to the general population, university students were significantly more likely to report being worried about/thinking about the coronavirus and to perceive the coronavirus as overrepresented in the media (all *p* < 0.001). University students reported a more supportive attitude toward vaccinations in general (students: *M* = 4.57, *SD* = 0.85; general population: *M* = 3.92, *SD* = 1.27) and a significantly higher vaccination intention (students: *n* = 4,438, 78.7%; general population: *n* = 635, 47.7%) than the general population (*p* < 0.001). Regression analyses revealed that in university students, vaccination intention was significantly predicted by not having children, a supporting attitude toward vaccinations in general, the belief that the coronavirus is overrepresented in the media, and less thinking about/worrying about the coronavirus (all *p* < 0.05). In the general population, vaccination intention was significantly associated with male gender, higher age, not having children, a supporting attitude toward vaccinations in general, and the belief that the coronavirus is overrepresented in the media (*p* < 0.05). The qualitative analysis among university students revealed that the most frequently stated positive aspect of the pandemic was to be more flexible due to digitalization (*n* = 1,301 statements, 22.2%) and the most frequently stated negative aspect was restriction in social life (*n* = 3,572 statements, 24.2%).

**Conclusion:**

The results indicate differences in the attitudes toward the pandemic between university students and the general population. In addition, differences regarding factors associated with vaccination intention were found in both samples. These results could be important to be considered when designing and targeting vaccination campaigns aiming at informing different population or age groups.

**Study registration:**

DRKS00022424.

## Introduction

On March 11^th^, 2020, the World Health Organization (WHO) proclaimed COVID-19 a pandemic, which emerged from China. Through several ways of transmission such as direct transmission, contact transmission and airborne transmission the virus has quickly spread throughout the world, affecting people of all generations (1). The governments mandated several measures to avoid the spread of SARS-CoV-2 in the absence of vaccines and specific effective therapy, until on December 21^st^, 2020, the first COVID-19 vaccine was authorized in Europe (2).

Vaccination is an important milestone toward achieving herd immunity and thus, protecting populations. However, despite the availability of vaccines, the COVID-19 pandemic is expected to continue, affecting societies worldwide, due to a lack of international vaccine distribution as well as vaccine hesitancy among the population (3). In 2019, the WHO identified vaccine hesitancy as one of the top global health threats (4). Vaccine hesitancy may be defined as a low vaccination intention, specifically a “delay in acceptance or refusal of vaccination despite availability of vaccination services” (5). Reasons for vaccination hesitancy might include (amongst others) a low perception of disease risk, restricted vaccine affordability, as well as general lack of trust and doubts about the efficiency and safety of the vaccine, and a belief to be already immunized (6). Understanding factors influencing COVID-19 vaccination intention still plays an important role to end or limit the COVID-19 pandemic. Studies have reported significant differences regarding the COVID-19 vaccination intention between countries. Few countries, like Portugal, Malta or Denmark, have reached the WHO's declared goal of 80% vaccination coverage (7). Countries like Germany have failed to meet this target. A survey within the COVID-19 Snapshot Monitoring project (COSMO) in January, 2022, in Germany showed that among people who had not yet received a COVID-19 vaccination, 13% reported they were planning to receive a vaccination, 10% were unsure, 12% were hesitant, and 63% refused receiving a vaccination (8–10). Besides the vaccine hesitation the waning immunity after vaccination or infection and different protection rates of vaccines agains the novel Coronavirus variants are playing an important role in containment of COVID-19 (11, 12).

Regarding refusal of vaccination, female gender, lower education level, poor vaccination compliance in the past, no chronic physical conditions (except for hypertension), and lower perceived severity of COVID-19 showed the strongest associations, while age showed an inverted U-shaped relationship (10, 13). A recent systematic review and meta-analysis of 28 studies highlighted an increase in refusal of COVID-19 vaccines over time (14). Being female, younger age, lower income or education, and belonging to an ethnic minority group were found to be consistent sociodemographic predictors of a low vaccination intention (14). A cross-sectional study in five countries reported the following predictors of vaccine hesitancy using a machine learning model: paranoid pandemic-related concerns, vaccination conspiracy beliefs, a general conspiracy mentality, COVID-19 anxiety, high perceived risk of infection, low perceived social rank, lower age, lower income, and higher population density (15). Inversely, another study reported a positive association between trust in governments and acceptance of the vaccination (offered by the employer; (16). Furthermore, the role of media/social media has also been investigated: Vaccine hesitant/resistant respondents from the United Kingdom were found to consume more information about the COVID-19 pandemic from social media, but less information from newspapers, television, and radio (17). Overall, social environment factors play an important role in vaccine intention as well as perception of the pandemic (18).

University students are in general a vulnerable population (19–22) and have also been hit hard by the pandemic (23–25), but little is known about their attitudes toward the pandemic, and especially toward vaccination.

The estimated intention to receive a COVID-19 vaccination among the university student population differs between different studies and across countries. It appears that comparable to other populations, there is still a relevant proportion of university students who are hesitant or unsure about receiving a vaccination (26, 27). A study from Italy demonstrated that 14% of the university students showed low vaccination intention (28). Based on similar rates of vaccination intention between students in healthcare and non-healthcare curricula, the authors suggested that vaccination intention may be influenced by motivational and psychological factors, not only by the medical knowledge of students. Further, some studies found higher vaccine acceptance among students in Health Schools compared to other faculties (29). In this study, conspiracy beliefs and social media-based knowledge about COVID-19 vaccines were associated with a lower vaccination intention (29).

This study aimed to investigate (a) students' attitudes toward the pandemic, (b) potential differences in attitudes between university students and the general population, and (c) to exploratively examine predictors of vaccination intention in both, university students and the general population. To this end, data from two research projects were used. First, attitudes toward the COVID-19 pandemic in German university students were assessed within a cross-sectional and anonymous online survey. Second, data from a cross-sectional survey within the COSMO project in the German general population were analyzed.

## Methods

### Study sample and setting

Data from two cross-sectional research projects were used, comprising a sample of German university students and a sample of the German general population, respectively.

Regarding the sample of university students, a cross-sectional online survey was conducted in students of the University of Leipzig, Germany, between March and April 2021 [for details on study procedure see (24)]. The survey took place during the second pandemic lockdown, which was in force since November 2020, and due to high infection rates, harder measures had been imposed since December 2020. All students at the university (*N*= ~ 30,000) were invited *via* email and social media channels of the university to participate. The only inclusion criterion was current enrollment as a university student, with no exclusion criteria being applied. The Ethics Committee of the Medical Faculty of the University of Leipzig waived approval for this study because of anonymity of the survey (March 3^rd^, 2021). All participants provided informed consent prior to participation. The sample comprised *n* = 5,642 participants. In order to ensure comparability with the sample of the general population, *n* = 3 participants were excluded due to an age < 18 years, resulting in a final student sample of *N* = 5,639.

Regarding the sample of the general population, data from the COVID-19 Snapshot Monitoring project (COSMO) was used. COSMO is an ongoing, serial cross-sectional study in the German general population aged 18 to 74 years, aiming to assess the relations between risk perceptions, knowledge, public trust and protective behavior regarding COVID-19 (30). Participants were members of an ISO 26362:2009-compliant online panel (respondi.de, https://www.iso.org/standard/43521.html). They were compensated for participation by the data collection company at their usual rate. The quota samples match current distributions of the general population regarding age, gender, and residency in a German federal state. The cross-sectional online surveys started in March 2020 and have since been conducted weekly or bi-weekly. Participants were recruited *via* an external study sample provider, and informed consent was provided prior to study participation. Ethical approval was obtained from the University of Erfurt's institutional review board (#20200302/20200501).

For this analysis, data from the 29^th^ wave (assessed in December 2020) was used (8), since this wave contained the respective variables for comparison. The non-probabilistic quota sample representing the German adult general population for the characteristics age x sex x state consisted of *n* = 1,387 respondents in total. In order to ensure comparability with the student sample, *n* = 56 participants were excluded due to an age > 70 years, resulting in a final sample of *N* = 1,331.

### Measures

#### Sociodemographic information

Surveys in both university students and the general population, respectively, contained information on sociodemographic data (gender, age, relationship status, having underage children, education, and migration background). Further, the presence of experiences related to the pandemic (current or past infection with the coronavirus, infection and/or death due to an infection in the circle of acquaintance) and the presence of chronic somatic diseases were assessed.

##### Attitudes toward the pandemic in university students

In the student sample, participants were asked how their personal situation was affected by the pandemic and about their attitudes toward the pandemic using 13 items, rated on a 5-point Likert scale from 1 = “do not agree at all” to 5 = “agree completely” (see **Table 2** for detailed items).

Further, positive and negative aspects of the pandemic were assessed in free text format. The answers were not restricted in number of words.

##### Attitudes toward the pandemic in university students and the general population

In both samples, four items on attitudes toward and perceptions of the pandemic were assessed (i. e., thinking about, worrying about, and fearing the coronavirus, respectively, and media representation of the coronavirus), rated on 7-point Likert scales (for details see **Table 3**). Further, the self-reported likelihood of infection with the coronavirus was assessed. To ensure comparability between the samples, the likelihood of infection was recoded in both samples into a 3-point scale from 1 = “unlikely” to 3 = “likely.”

One item was used to assess the attitude toward vaccinations in general on a 5-point Likert scale from 1 = “rejecting” to 5 = “supporting” in both samples. Finally, vaccination intention regarding COVID-19 vaccination was assessed with one item in both samples (“If you had the possibility to receive a vaccination against COVID-19 in the next week, how would you decide?”), with answers being harmonized across samples to reflect a dichotomous answer format (yes/no).

### Statistical analyses

First, descriptive statistics on sociodemographic characteristics and experiences related to the pandemic in both samples were reported. Sample differences in these variables were examined using χ^2^ tests for all categorical dependent variables (gender, relationship status, having underage children, education, migration background, current or past infection with the coronavirus, infection in the circle of acquaintance, and death due to an infection in the circle of acquaintance) and Mann-Whitney *U* test for the continuous dependent variable (age), due to non-normal distribution (as indicated by Shapiro-Wilks test, *p* < 0.05).

Second, to analyze students' attitudes toward the pandemic, descriptive statistics on 13 items assessing personal attitudes were reported. Further, the qualitative data of the free text fields of positive and negative aspects of the pandemic were analyzed using MAXQDA qualitative software (version 2022.0.0) to manage and code the textual data. Based on Mayrings approach of the summarizing content analysis (31), a coding dictionary was developed to analyze the answers, separately for the positive and negative aspects, respectively. The aim was to develop as few codes as possible, but as many as necessary to represent every free text statement in the coding. One author coded all qualitative data with the final coding manual. To ensure validity of the coding manual, inter-rater reliability was estimated: A randomly selected subset (25%) of the qualitative data of the positive aspects was coded by a second researcher unfamiliar with the project, and both ratings were then compared (32). The resulting estimated inter-rater reliability of κ = 0.80 is based on a mean-rating (*k* = 2), absolute-agreement, 2-way mixed-effects model. This estimation is indicative of a very good reliability (33).

Third, differences in attitudes between university students and the general population were analyzed. Group differences in continuous dependent variables (four items on attitudes toward the pandemic, self-reported likelihood of infection, attitude toward vaccinations in general) were analyzed using Mann-Whitney *U* tests, due to non-normal distribution of all dependent variables (as indicated by Shapiro-Wilks tests, all *p* < 0.05). Differences on the categorical dependent variable (vaccination intention) were computed using a χ^2^ test.

Finally, two multivariable logistic regression analyses were performed to examine predictors of vaccination intention (dependent variable) in university students and the general population, respectively, separately in each sample. The following variables were included as independent (predictor) variables: gender, age, relationship status, having underage children, education, migration background, chronic disease, likelihood of infection, attitude toward vaccinations in general, and four items on attitudes toward the pandemic (i. e., thinking about, worrying about, and fearing the coronavirus, respectively, and media representation of the coronavirus). Data were checked for outliers. Further, correlations between predictors were low (*r* < 0.80), indicating that multicollinearity was not a confounding factor.

To ensure comparability between the samples regarding gender, people with diverse gender in the student sample (*n* = 84, 1.5%) were excluded from the analysis on group differences in gender and from the multivariable logistic regression analysis, as the survey in the general population only assessed male and female, but not diverse gender.

To estimate effect sizes for χ^2^ tests, the φ coefficient was used, with φ = 0.10 indicating a small, φ = 0.30 a medium, and φ = 0.50 a large effect (34). Effect sizes for Mann-Whitney *U* tests were interpreted as small, *r* < 0.30, medium, *r* < 0.50, and large, *r* > 0.50 (34). In the logistic regression analyses, the amount of explained variance as indicated by *Nagelkerke's R*^2^ was interpreted as small, *R*^2^ > 0.20, medium, *R*^2^ > 0.40, and large, *R*^2^ > 0.50 (35). Statistical analyses were performed using IBM SPSS Statistics version 27.0. A two-tailed α = 0.05 was applied to statistical testing. In the case of missing values, participants with missing values were excluded from the respective analyses. Descriptive statistics were reported including only valid cases.

## Results

### Sample characteristics

The student sample comprised *n* = 3,914 (70.5%) female and *n* = 1,641 (29.5%) male participants with a mean age of 23.47 years (*SD* = 4.46, range 18–70 years), while the sample of the general population consisted of *n* = 669 (50.3%) female and *n* = 662 (49.7%) male participants with a mean age of 44.22 years (*SD* = 15.03, range 18–70 years; see Table 1). Regarding relationship status, in the student sample *n* = 2,708 (48.0%) stated being in a relationship, while the sample of the general population consisted of *n* = 930 (69.9%) participants in a relationship.

**Table 1:** Sociodemographic characteristics and group differences between university students and the general population.

**Variable**	**University students** **(*n* = 5,639)**	**General population** **(*n* = 1,387)**	**Test**	***p***	**Effect size**
**Gender, *n* (%)**			χ^2^ (1.6886) = 196.75	**<0.001**	φ = 0.17
Female	3.914 (70.5)	669 (50.3)			
Male	1.641 (29.5)	662 (49.7)			
Age, *M* (*SD*)	23.47 (4.46)	44.22 (15.03)	*U* = 768,611.50	**<0.001**	*r* = 0.54
**Relationship status, *n* (%)**			χ^2^ (1.6970) = 206.03	**<0.001**	φ = 0.17
In a relationship	2.708 (48.0)	930 (69.9)			
Single	2.931 (52.0)	401 (30.1)			
Children under 18, *n* (%)	237 (4.2)	391 (29.4)	χ^2^ (1.6970) = 832.37	**<0.001**	φ = 0.35
Higher education (≥ 12 years), *n* (%)	5.278 (93.6)	744 (55.9)	χ^2^ (1.6970) = 1.302.43	**<0.001**	φ = 0.43
Migration background, *n* (%)	647 (11.5)	213 (16.1)	χ^2^ (1.6965) = 20.90	**<0.001**	φ = 0.06
Current or past infection with COVID-19, *n* (%)	263 (4.7%)	46 (3.5%)	χ^2^ (1.6970) = 3.71	0.054	φ = 0.02
Knowing someone with COVID-19 infection, *n* (%)	4.304 (76.3%)	491 (36.9%)	χ^2^ (1.6970) = 780.10	**<0.001**	φ = 0.34
Knowing someone who died due to COVID-19, *n* (%)	907 (21.1%)	113 (23.0%)	χ^2^ (1.4795) = 0.99	0.323	φ = 0.01

Calculation of % from valid cases. Bold values indicate statistical significance at the *p* < 0.05 level.

Significant differences between both samples (small to medium effects) were found for all variables except for current or past infection with COVID-19 and knowing someone who died due to COVID-19, respectively (both *p* > 0.05; see Table 1). Specifically, in comparison with the general population, the sample of university students consisted of significantly more females, reported a lower age, was less likely to be in a relationship, have underage children or report a migration background. Further, students had a significantly higher educational level, as expected. Finally, the percentage of participants knowing someone with a COVID-19 infection was significantly higher among students than among the general population.

#### Attitudes toward the pandemic in university students

When asked about their attitudes toward the pandemic, students tended to be rather worried because of COVID-19 (*M* = 3.77, *SD* = 1.04), while still being optimistic about surviving the crisis unharmed (*M* = 3.49, *SD* = 1.01; see Table 2). Further, while generally supporting the government-mandated measures (*M* = 3.83, *SD* = 1.02), participants also indicated that they felt restricted by them (*M* = 3.56, *SD* = 1.11). The results further imply that students viewed themselves as particularly hit hard by the corona crisis in general (*M* = 3.73, *SD* = 1.01) and by the measures to reduce the crisis (*M* = 3.69, *SD* = 1.05). Overall, participants did not agree with the statements that the pandemic is part of a conspiracy (*M* = 1.14, *SD* = 0.51) and that they feel responsible for the corona crisis (*M* = 1.43, *SD* = 0.80).

**Table 2:** Attitudes toward the pandemic in university students (*N* = 5,639).

**Item**	***M (SD)***
I am worried because of COVID-19.	3.77 (1.04)
I personally feel in danger because of COVID-19.	2.77 (1.08)
I am particularly at risk from the coronavirus due to existing medical conditions.	1.57 (1.03)
I fully support government measures to slow down the spread of the coronavirus.	3.83 (1.02)
I feel severely restricted by the government measures to slow down the coronavirus.	3.56 (1.11)
I think the general fear of the coronavirus is exaggerated.	2.02 (1.06)
Government measures to slow down the spread of the virus are excessive, they do more harm than good.	2.19 (1.08)
I am optimistic that I will survive the corona crisis unscathed.	3.49 (1.01)
Students are particularly hit hard by the corona crisis.	3.73 (1.01)
The measures to reduce the crisis hit students particularly hard.	3.69 (1.05)
Overall, it is good for me that I do not have to go out as much and have less contact with other people.	1.85 (1.05)
The pandemic is part of a larger conspiracy.	1.14 (0.51)
I feel responsible for the corona crisis.	1.43 (0.80)

All items were assessed on 5-point answer scales from 1 = “do not agree at all” to 5 = “agree completely.”

Students had also been asked in free text format about positive and negative aspects of the pandemic. The results of the qualitative analysis revealed that the most frequent positive aspects among the *N* = 5,858 statements were (in descending order): (1) flexibility due to more digitalization (e. g., online lectures; *n* = 1,301, 22.2%), (2) more intense social contacts (*n* = 773, 13.2%), (3) more time for yourself (*n* = 488, 8.3%), (4) deceleration, calm, and less stress (*n* = 451, 7.7%), and (5) more free time due to less commuting time (*n* = 380, 6.5%).

The most frequently reported negative aspects of the pandemic among *N* = 14,792 statements in total were (in descending order): (1) restrictions in social life (*n* = 3,572, 24.2%), (2) restrictions in use of leisure time (*n* =1,137, 7.7%), (3) loss of daily structure and difficulties due to being home alone all day (*n* = 834, 5.6%), (4) negative economic and occupational impact (*n* = 785, 5.3%), and (5) challenges of home office and remote working or learning (*n* = 772, 5.2%).

#### Attitudes toward the pandemic in university students and the general population

In comparison to the general population, students were significantly more likely to report being worried about and thinking about the coronavirus, and to perceive the coronavirus as overrepresented in the media (all *p* < 0.001, small effects; see Table 3). No significant sample differences emerged regarding fear of the virus (*p* > 0.05).

**Table 3:** Differences in attitudes toward the pandemic between university students and the general population.

	**University students** **(*n* = 5,639)**	**General population** **(*n* = 1,387)**	**Test**	***p***	**Effect size**
**Item/variable**	**M (SD)**	**M (SD)**			
The coronavirus is…					
… something I permanently think about / hardly ever think about^a^	2.95 (1.39)	3.76 (1.56)	*U* = 2,607,067.50	**<0.001**	*r* = 0.21
… frightening / not frightening^a^	3.62 (1.63)	3.68 (1.75)	*U* = 3,696,505.00	0.386	*r* = 0.01
… overrepresented in the media / not represented enough in the media^a^	3.18 (1.17)	3.39 (1.57)	*U* = 3,445,732.50	**<0.001**	*r* = 0.06
… something I worry about / do not worry about^a^	2.60 (1.52)	3.09 (1.72)	*U* = 3,131,671.00	**<0.001**	*r* = 0.12
Likelihood of infection	1.86 (0.71)	1.90 (0.82)	*U* = 3,697,748.00	0.369	*r* = 0.01
Attitude toward vaccinations in general	4.57 (0.85)	3.92 (1.27)	*U* = 2,553,285.00	**<0.001**	*r* = 0.24
	***n* (%)**	***n* (%)**			
Vaccination intention	4.438 (78.7)	635 (47.7)	χ^2^ (1.6970) = 522.18	**<0.001**	φ = 0.27

a Items were assessed on scales from 1 to 7, with two verbal anchors for 1 and 7, respectively. Bold values indicate statistical significance at the *p* < 0.05 level.

Further, samples did not differ in the perceived likelihood of infection (*p* > 0.05). However, students reported a more supportive attitude toward vaccinations in general and a significantly higher vaccination intention than the general population (all *p* < 0.001, small effects).

#### Predictors of COVID-19 vaccination intention in university students and the general population

Both logistic regression models in university students and the general population, respectively, were statistically significant (all *p* < 0.001), resulting in a large amount of explained variance in university students (*Nagelkerke's R*^2^ = 0.55) and a medium amount of explained variance in the general population (*Nagelkerke's R*^2^ = 0.42; see Table 4). In university students, vaccination intention was significantly predicted by not having underage children (*p* = 0.016), a supporting attitude toward vaccinations in general, the belief that the coronavirus is overrepresented in the media, and less thinking about and worrying about the coronavirus (all *p* < 0.001). In the general population, vaccination intention was significantly predicted by male gender (*p* < 0.001), higher age (*p* = 0.004), not having underage children (*p* = 0.016), a supporting attitude toward vaccinations in general, and the belief that the coronavirus is overrepresented in the media (all *p* < 0.001).

**Table 4:** Predictors of vaccination intention in university students and the general population.

**Predictor variable**	**University students**				**General population**			
	**(*n* = 5,469)^a^**				**(*n* = 1,260)^a^**			
	***B***	***SE***	***p***	**OR [95% CI]**	***B***	***SE***	***p***	**OR [95% CI]**
Gender	−0.16	0.11	0.133	0.85 [0.69; 1.05]	−0.89	0.14	**<0.001**	0.41 [0.31; 0.55]
Age	0.02	0.01	0.144	1.02 [0.99; 1.04]	0.02	0.01	**0.004**	1.02 [1.01; 1.03]
Relationship status	0.08	0.10	0.429	1.08 [0.90; 1.30]	−0.03	0.16	0.863	0.97 [0.72; 1.32]
Children under 18	−0.58	0.24	**0.016**	0.56 [0.35; 0.90]	−0.38	0.16	**0.016**	0.68 [0.50; 0.93]
Higher education	−0.13	0.21	0.520	0.88 [0.58; 1.31]	0.03	0.15	0.842	1.03 [0.77; 1.38]
Migration background	0.01	0.15	0.957	1.01 [0.76; 1.34]	0.22	0.21	0.288	1.24 [0.83; 1.86]
Chronic disease	−0.06	0.13	0.626	0.94 [0.73; 1.21]	0.08	0.16	0.626	1.08 [0.80; 1.46]
Likelihood of infection	0.04	0.07	0.603	1.04 [0.91; 1.18]	0.07	0.09	0.476	1.07 [0.89; 1.28]
Attitude toward vaccinations	2.01	0.07	**<0.001**	7.44 [6.52; 8.50]	1.05	0.08	**<0.001**	2.86 [2.46; 3.32]
Thinking about coronavirus	−0.16	0.04	**<0.001**	0.85 [0.79; 0.92]	−0.02	0.06	0.744	0.98 [0.87; 1.10]
Worrying about coronavirus	−0.22	0.04	**<0.001**	0.81 [0.74; 0.88]	−0.06	0.06	0.343	0.94 [0.83; 1.07]
Fear of coronavirus	−0.03	0.04	0.479	0.97 [0.89; 1.05]	−0.07	0.06	0.249	0.93 [0.82; 1.05]
Media representation of coronavirus	0.40	0.04	**<0.001**	1.50 [1.38; 1.63]	0.24	0.05	**<0.001**	1.27 [1.15; 1.40]
Constant	−7.76	0.55	<0.001	0.00	−4.98	0.60	<0.001	0.01
χ^2^	χ^2^ (13) = 2385.84, *p* < 0.001				χ^2^ (13) = 482.42, *p* < 0.001			
*R^2^* (Cox-Snell / Nagelkerke)	0.35 / 0.55				0.32 / 0.42			

a Reduced sample sizes due to missing values. Coding for gender: 0 = male, 1 = female. OR, odds ratio; CI, confidence interval.

Bold values indicate statistical significance at the *p* < 0.05 level.

## Discussion

This study examined attitudes toward the pandemic and predictors of COVID-19 vaccination intention in university students and the general population. The results showed significant differences in attitudes toward the pandemic between both samples. Further, besides negative aspects, many of the university students reported various positive aspects of the pandemic. The results also indicate that predictors of vaccination intention in university students and the general population are overall similar, despite slight differences.

Regarding their attitudes toward the pandemic, university students in the present study tended to be worried and frightened because of the pandemic. Further, they were significantly more likely to be worried and think about the coronavirus in comparison to the general population. Only few previous studies focused on understanding the attitudes and beliefs of university students regarding the COVID-19 pandemic. One of these studies reported that 38% of university students were worried about the coronavirus, and 44% of them stated to fear an infection (36). In addition, a recent meta-analysis reported that students experienced a moderate level of fear concerning the pandemic (37), which is in accordance with our findings. Overall, these findings emphasize that university students are more vulnerable to the pandemic situation and the side effects of control measures compared to the general population (25).

To our knowledge this is the first study conducted in university students examining positive and negative aspects of the pandemic assessed in free text format. Despite the frequently mentioned negative aspects on various platforms (e. g., on social media or in the news), such as restrictions in social life and leisure time, university students in this study also reported various positive aspects like flexibility due to more digitalization (22.2%), more intense social contacts (13.2%), and more time for themselves (8.3%). Furthermore, a certain percentage of students (7.7 %) described being calm and less stressed as a positive aspect of the pandemic. This result is in line with a study reporting that a “calmer life” was one of the most common positive effects reported (38).

Students in this study showed a significantly higher vaccination intention (78.7%) than the general population (47.7%). In contrast to this finding, earlier studies reported a higher vaccine hesitancy in young people compared to older populations (14, 39). However, an inverted-U-shaped relationship between age and anti-COVID vaccination behavior was also reported, which might explain our findings (13). Important to highlight here again that (given the nature of both samples) there is an age difference between the sample of university students and the sample of the general population. It might be that some of the differences in vaccination intention could also be explained by this age difference. Further studies among young people not being university students would be needed to clarify this.

In our study, vaccination intention in both university students and the general population was significantly predicted by not having underage children, a supporting attitude toward vaccinations in general, and the belief that the coronavirus is overrepresented in the media. In addition, less thinking about and worrying about the coronavirus significantly predicted a higher vaccination intention in students, while male gender and higher age were predictors in the general population. The results of the regression analyses were mostly in line with previous findings. Regarding the general population, studies also showed an association of female gender and younger age with a low vaccine intention (14, 39, 40). However, gender was not a significant predictor in university students in our study. In line with this, there are also studies suggesting that gender does not play a role in self-reported willingness to receive a COVID-19 vaccine (41). Furthermore, our results showed no predictive effect of migration background and education on vaccine intention in both groups, which was also reported by other studies (14, 39). Earlier studies also indicated that having school-age children was related with refusal of COVID-19 vaccine (42), which was in line with our results. However, when interpreting the results on the association between not having underage children and vaccination intention, the uneven distribution of having children in both samples (as would be expected from the nature of the samples) has to be considered. Further, as would be expected, positive attitudes toward vaccinations were associated with vaccine intention in both groups. On the other hand, the belief that the coronavirus is overrepresented in the media was positively associated with vaccine intention in both groups, which was unexpected since previous studies emphasized a positive association between vaccine hesitancy and higher social media consumption (43, 44). A potential explanation for this somewhat unintuitive finding could be that people believe in the vaccination to be a secure and safe way to combat the pandemic and end the “over”-representation in the media and move back to daily life with no or at least less restrictions.

Strengths of this study include the large sample sizes for both samples of university students and the general population, respectively, and the mixed-methods approach including quantitative and qualitative methods. The student sample included university students from all faculties of the University of Leipzig, which is an important strength considering the fact that most previous studies focused mainly on students in healthcare settings. Additionally, both surveys were conducted during the second peak time of the pandemic in Germany, with higher mortality and morbidity rates, which makes the findings particularly relevant. Nevertheless, although very close in time, the time points of the surveys were not identically (which was due to fact that not all waves of the general population survey contained the respective variables for comparison). Hence, it were different time points in seasonality of SARS-CoV-2 transmission, vaccine availability in Germany, case numbers, ICU occupancy and dominant SARS-CoV-2 variants, which might additionally influence respondents' attitudes and answers in the surveys. Other limitations might also be considered when interpreting the results. First, no causal relationships can be determined due to the cross-sectional study design. Second, only students of one German university were contacted for the anonymous survey, which might lead to underrepresentation of the attitudes and vaccine intention among students in other regions of Germany. Third, the nature of data collection might have resulted in a selection bias. As vaccine intention varies in each country, the measurement implemented by governments also vary (45, 46). Trust in government policies as well as healthcare sector are playing important roles as predictors of vaccine intention (47). Also, other correlates of vaccine hesitancy such as trust in science in vaccine development and negative perceptions of safety were reported as significant predictors of vaccine hesitancy in different investigations (48).

In conclusion, the results of this study might be important to be considered when designing and targeting vaccination campaigns to university students as opposed to messages to the general public. Specifically, it is of great importance to include university students in the COVID-19 vaccination program considering that they are an important risk group due to their vulnerability to an infection with the coronavirus and transmission-associated behaviors. The results on the attitudes of students and the general population about the pandemic in general and about the COVID-19 vaccine in particular may be useful to support health engagement and plan future management of public health strategies. Additionally, implementing more digital platforms for a low-threshold access to reliable information on the COVID-19 vaccine may reduce vaccine hesitancy among university students and also the general population. Further, to our knowledge this is the first study investigating not only negative, but also positive aspects of the pandemic reported by university students. It is of great importance to identify positive aspects of the pandemic and related restrictions to find ways to promote community resilience.

## Data availability statement

The raw data supporting the conclusions of this article will be made available by the authors, without undue reservation.

## Ethics statement

The studies involving human participants were reviewed and approved by University Leipzig, Ethical Committee of the Medical Faculty. The patients/participants provided their written informed consent to participate in this study.

## Author contributions

EK and CR-K conceptualized the study and constructed the questionnaires. SB, ED-S, and EK performed data analyses and drafted the initial version of the manuscript. SM, FD, LH, and CR-K critically reviewed and revised the manuscript. All authors have read and approved the content of the final manuscript.
